# LC–MS/MS based 25(OH)D status in a large Southern European outpatient cohort: gender- and age-specific differences

**DOI:** 10.1007/s00394-018-1803-1

**Published:** 2018-08-07

**Authors:** Silvia Giuliani, Verena Barbieri, Angela Maria Di Pierro, Fabio Rossi, Thomas Widmann, Manuela Lucchiari, Irene Pusceddu, Stefan Pilz, Barbara Obermayer-Pietsch, Markus Herrmann

**Affiliations:** 1 Department of Clinical Pathology, District Hospital Bolzano, Bolzano, Italy; 2 Interdisciplinary Medical Research Center (IMREST), Bolzano, Italy; 3 39100 Bolzano, Italy; 4 Department of Oncologic Rehabilitation, Asklepios Clinic Triberg, Triberg, Germany; 5 Division of Endocrinology and Diabetology, Department of Internal Medicine, Medical University of Graz, Graz, Austria; 6 Clinical Institute for Medical and Chemical Laboratory Diagnostics, Medical University of Graz, Auenbruggerplatz 15/1, 8036 Graz, Austria

**Keywords:** 25(OH)D, PTH, Mass spectrometry, Data mining

## Abstract

**Background:**

Developed countries have a high prevalence of vitamin D deficiency. In previous studies, 25(OH)D was predominantly measured by immunoassays. The present study assessed serum 25(OH)D in a very large Southern European outpatient cohort by liquid chromatography tandem mass spectrometry (LC–MS/MS).

**Materials and methods:**

74,235 serum 25(OH)D results generated under routine conditions between 2015 and 2016 were extracted from the laboratory information system of the Department of Clinical Pathology at Bolzano Hospital (Italy). In 3801 cases, parathyroid hormone (PTH) was requested in parallel. Serum 25(OH)D was measured by a NIST-972 aligned commercial LC–MS/MS method. The distribution of serum 25(OH)D concentrations in males and females of different age groups, the prevalence of 25(OH)D_2_ and seasonal variability were studied.

**Results:**

The average 25(OH)D concentration in the entire cohort was 68.6 nmol/L (7.5–1880 nmol/L). Females had a 7 nmol/L higher average 25(OH)D concentration than males, which increased significantly with age. 37.9 and 28.3% of males and females, respectively, had a deficient 25(OH)D concentration of < 50 nmol/L. 620 samples (0.84%) had measureable amounts of 25(OH)D_2_. In samples with a normal PTH, 25(OH)D was 11 nmol/L higher than in the entire cohort. Seasonal variation ranged between 20 and 30% and was most pronounced in young individuals. 25(OH)D_2_ remained constant throughout the year.

**Conclusion:**

Average serum 25(OH)D in South Tyrol is higher than in other parts of Europe. 25(OH)D and PTH show a continuous inverse relationship. Seasonal variation of serum 25(OH)D is an important aspect in young and middle-aged adults, but becomes less relevant in elderly subjects. 25(OH)D_2_ is of minor practical importance in South Tyrol.

**Electronic supplementary material:**

The online version of this article (10.1007/s00394-018-1803-1) contains supplementary material, which is available to authorized users.

## Introduction

Vitamin D deficiency is a risk factor for osteomalacia, osteoporosis and fractures [1]. Multiple studies have demonstrated that vitamin D deficiency is also associated with an increased risk to develop other diseases, such as cancer [1], diabetes [2, 3], sarcopenia [4], cardiovascular [5] and autoimmune disease [6]. The increased awareness of the high prevalence of vitamin D deficiency has triggered an exponential increase in 25(OH)D testing.

The appropriate interpretation of serum 25(OH)D results is a matter of ongoing debate. Consequently, the recommended 25(OH)D cut-offs vary between different scientific bodies [7–10]. Age or gender specific cut-offs are not suggested by any guideline although several studies suggest that physiologic 25(OH)D requirements may vary with age, gender and ethnicity [11–15]. Interpretation of 25(OH)D results is further complicated by a variable accuracy of 25(OH)D assays and a strong seasonal variability of up to 20% [16–19]. Automated immunoassays represent the most widely used method in daily practice. However, the analytical performance of these assays varies substantially [16–19]. In particular, they vary in their efficiency to separate 25(OH)D from its carrier proteins and are often subject to interferences, such as high concentrations of 25(OH)D_2_ or heterophile antibodies. LC–MS/MS is the most accurate method for the measurement of 25(OH)D, but is mainly used in research laboratories.

Previous studies have shown a high prevalence of vitamin D deficiency in virtually all developed countries around the globe [20]. However, many of these studies have used immunoassays for the measurement of 25(OH)D [20]. Considering the analytical performance of 25(OH)D immunoassays, the trueness of results from previous epidemiologic studies is questionable.

Meanwhile, a reference method and a standard reference material (NIST SRM 972a) for the measurement of 25(OH)D have been developed [21, 22]. In addition, the Vitamin D Standardization Program (VDSP) has been launched with the goal to improve accuracy and comparability of analytical methods. However, until today, this initiative has only partially resolved the analytical issues of automated 25(OH)D immunoassays. Recently, Cashman et al. performed a reanalysis of previously measured samples with a standardized LC–MS/MS method [20]. The results show substantial over- or underestimation of immunoassays. For example, in a German adult survey, the pre-standardization prevalence of 25(OH)D serum levels < 30 nmol/L was 25.9% and decreased to 15.2% after standardization. In view of the analytical issues of immunoassays and the very low number of studies that have used higher order methods for the quantitation of 25(OH)D, the vitamin D status of most populations is not well described. Moreover, it remains unclear if the cut-off values proposed by various scientific bodies around the globe are applicable when a NIST 972a aligned LC–MS/MS method is used to measure 25(OH)D.

The present study aimed to describe the 25(OH)D status in South Tyrol, a mountainous region in the Southern European Alps using a rigorously controlled, NIST 972a aligned LC–MS/MS. In particular, we studied the distribution of serum 25(OH)D concentrations in males and females of different age groups, the prevalence of measurable amounts of 25(OH)D_2_ and seasonal variability.

## Materials and methods

### Study design

We analyzed retrospectively all serum 25(OH)D results from outpatients that were generated between January 1, 2015 and December 31, 2016 at the Central Laboratory of Clinical Pathology at the Bolzano Hospital (Italy). Out of 74,235 samples 3801 cases were identified where PTH was requested at the same occasion. Results were used to study the distribution of serum 25(OH)D concentrations in males and females of different age groups, the prevalence of measurable amounts of 25(OH)D_2_ and seasonal variability.

All measurements were performed under routine circumstances. Samples were collected in serum tubes with clot activator. As per routine procedure, samples were centrifuged upon arrival in the lab and stored at 4 °C until measurement. 25(OH)D_3_ and 25(OH)D_2_ were quantitated separately. Results from subjects < 18 years were excluded from the analysis. The study was approved by the local Ethics Committee.

### Biochemical analyses

25(OH)D analyses by LC–MS/MS were performed as part of our routine clinical work with a commercial kit from Recipe (Munich, Germany) that is traceable to the NIST 972a standard reference material. The method is continuously controlled by daily internal and monthly external quality controls provided by the Royal Australian College of Pathologists Australasia Quality Assurance Program (RCPAQAP). Samples were measured on a Shimadzu 8040 LC–MS/MS instrument coupled to a UHPLC system (Nexera, Shimadzu). Sample preparation consisted in protein precipitation with zinc sulfate, the addition of internal standard (d6 25[OH]D_3_) eluted in methanol and subsequent online solid phase extraction (Recipe, code MS7030). The method was calibrated with ClinCal Serum Calibrators from RECIPE (Munich, Germany) and allow an equimolar detection of the two major 25(OH)D species, 25(OH)D_3_ and 25(OH)D_2_. According to manufacturer’s declaration for 25(OH)D_3_, the assay is linear between 7.5 and 375 nmol/L, limit of detection(LoD) is 1.7 nmol/L and limit of quantification (LoQ) is 7.5 nmol/L. For 25(OH)D_2_, the assay is linear between 3.5 and 605 nmol/L, LoD is 1.0 and LoQ is 3.5 nmol/L. Imprecision is < 8.8% and < 8.7 for 25(OH)D_3_ and 25(OH)D_2_, respectively. In our laboratory, the long-term total imprecision was < 7.3% at high and low levels. For statistical purposes, samples below the LoQ have been assigned a value of 3.75 nmol/L, which is half way between 0 and 7.5 nmol/L.

PTH was measured in lithium-heparin plasma using the PTH STAT assay from Roche Diagnostics (Mannheim, Germany) on a Cobas 8000 auto-analyzer. Total imprecision was 3.8 and 2.6% at concentrations of 13.6 and 361.3 pg/mL, respectively. The reference range provided in the manufacturers package insert is 15–65 pg/mL.

### Statistical analysis

We calculated the following descriptive statistics: mean, median 2.5th, 5th 95th 97.5th percentile for total 25(OH)D, 25(OH)D_3_ and 25(OH)D_2_. Subgroups were formed on the base of gender, age, season and serum PTH. Differences between groups were explored using the Mann–Whitney (2 groups) or the Kruskal–Wallis (> 2groups) test. A *p* value of < 0.01 was considered significant. For post hoc analysis, a Wilcoxon test was used.

Seasonal variation was explored using the reference limit estimator (version 17.10.2015) developed by the German Society of Clinical Chemistry and Laboratory Medicine (DGKL), based on R version 3.1.0. In addition, 25(OH)D results were plotted against the date of analysis. A GAM (generalized additive model) model was used to smoothen the median over time and to calculate 95% CI for it. Using median values per month, a weighted spline-based smoothing function is applied.

Statistical analyses were performed with Microsoft Excel 2010 (Microsoft, Redmond, WA,USA) and Medcalc v17.4.4 software (Belgium).

## Results

### General characteristics of the cohort

From all 74,235 serum 25(OH)D results, 18,811 (25.3%) were from males and 55,424 (74.7%) from females. The mean age of all subjects was 59.3 years (range 18–104 years). Serum 25(OH)D was predominantly tested in individuals between 41 and 80 years of age (75.4%). Subjects between 19 and 40 years accounted for only 14.6% of all requests. During summer/autumn, the number of requests was lower than in the winter/spring period: 35.316 requests (47.6%) vs. 38.919 requests (52.4%).

### Global serum 25(OH)D status in the South Tyrolean population

The average 25(OH)D concentration in the entire cohort was 68.6 nmol/L (range 7.5–1880 nmol/L). The 2.5th and 97.5th percentile spanned a rather wide range from 12 to 159 nmol/L (Table 1). In 0.5% of all samples, 25(OH)D was below 7.5 nmol/L, the LoQ of our method. Subjects with non-quantifiable 25(OH)D had a mean age of 68 years and were predominantly females. The prevalence of non-quantifiable 25(OH)D concentrations increased with age: 19–40 years: 9.5%, 41–60: 23.6%, 61–80: 32.5% and > 80: 34.4%, respectively. Table 2 shows vitamin D distribution according to different 25(OH)D cut-off levels.

**Table 1:** 25(OH)D concentration in 74,235 serum samples from South Tyrol—descriptive statistics

Sex	All		19–40 years		41–60 years		61–80 years		> 80 years	
	M (*n* = 18,811)	F (*n* = 55,424)	M (*n* = 2684)	F (*n* = 7900)	M (*n* = 6437)	F (*n* = 20,780)	M (*n* = 7703)	F (*n* = 21,076)	M (*n* = 1987)	F (*n* = 5668)
25(OH)D, nmol/L—entire cohort										
Median	60	67*	57	60*	60	67*	57	70*	52	67*
Mean ± SD	63.6 ± 39.9	70.2 ± 38.6	64 ± 44.6	66.0 ± 41.2	66.4 ± 42.5	70.1 ± 48.6	62.8 ± 36	72.6 ± 36.6	57.5 ± 36.2	68 ± 41.5
2.5th–97.5th percentiles	12–150	12–152	15–152	15–150	15–162	17–152	12–142	15–152	10–137	7–159
5th–95th percentiles	17–125	17–132	20–127	20–127	20–135	22–130	17–122	20–132	12–120	10–137

Sex	M (*n* = 548)	F (*n* = 1684)	M (*n* = 64)	F (*n* = 133)	M (*n* = 216)	F (*n* = 654)	M (*n* = 206)	F (*n* = 688)	M (*n* = 62)	F (*n* = 209)
25(OH)D, nmol/L—subjects with normal PTH										
Median	70	82*	63.5	75*	67	75*	77	86*	72.5	90*
Mean ± SD	77.1 ± 43.3	85.9 ± 41.22	69.6 ± 34.5	80.1 ± 41.49	78.7 ± 48.8	80.6 ± 42.3	78.3 ± 40.1	89.47 ± 39.2	75.1 ± 37.6	94.3 ± 41.56
2.5th–97.5th percentile	19–185	22–187	22–147	22–194	18–199	22–182	20–152	23–179	17–153	16–205
5th–95th percentile	22–152	27–157	22–132	28–179	24–184	27–152	23–135	27–155	20–144	32–159

*p < 0.001 vs. males

**Table 2:** Vitamin D distribution according to different 25(OH)D cut-off levels

	20 nmol/L		30 nmol/L		40 nmol/L		50 nmol/L		60 nmol/L		75 nmol/L		100 nmol/L		125 nmol/L	
	% below	% above	% below	% above	% below	% above	% below	% above	% below	% above	% below	% above	% below	% above	% below	% above
25(OH)D cut-off level																
Entire cohort	6.8	93.2	14.5	85.5	23.4	76.6	33.3	66.7	45	55	63.1	36.9	85	15	94.1	5.9
M	2.0	23.3	4.5	20.8	7.4	18	10.3	15	13.4	11.9	17.7	7.6	22	3	24.1	1.3
F	4.8	69.9	10	64.7	16	58.6	23	51.7	31.6	43.1	45.4	29.3	63	12	70	4.6

### Prevalence of measurable serum 25(OH)D_2_ in the South Tyrolean population

In 620 samples, 25(OH)D_2_ was detected in quantifiable concentrations. This accounts for 0.84% of the entire cohort. In subjects between 61 and 80 years, 25(OH)D_2_ was found most frequently. The average concentration in males and females was 13.5 and 17.0 nmol/L, respectively, and did not change with age (differences between age groups were not significant, supplementary table 1).

### Differences in serum 25(OH)D status based on age and gender

**Fig. 1:**
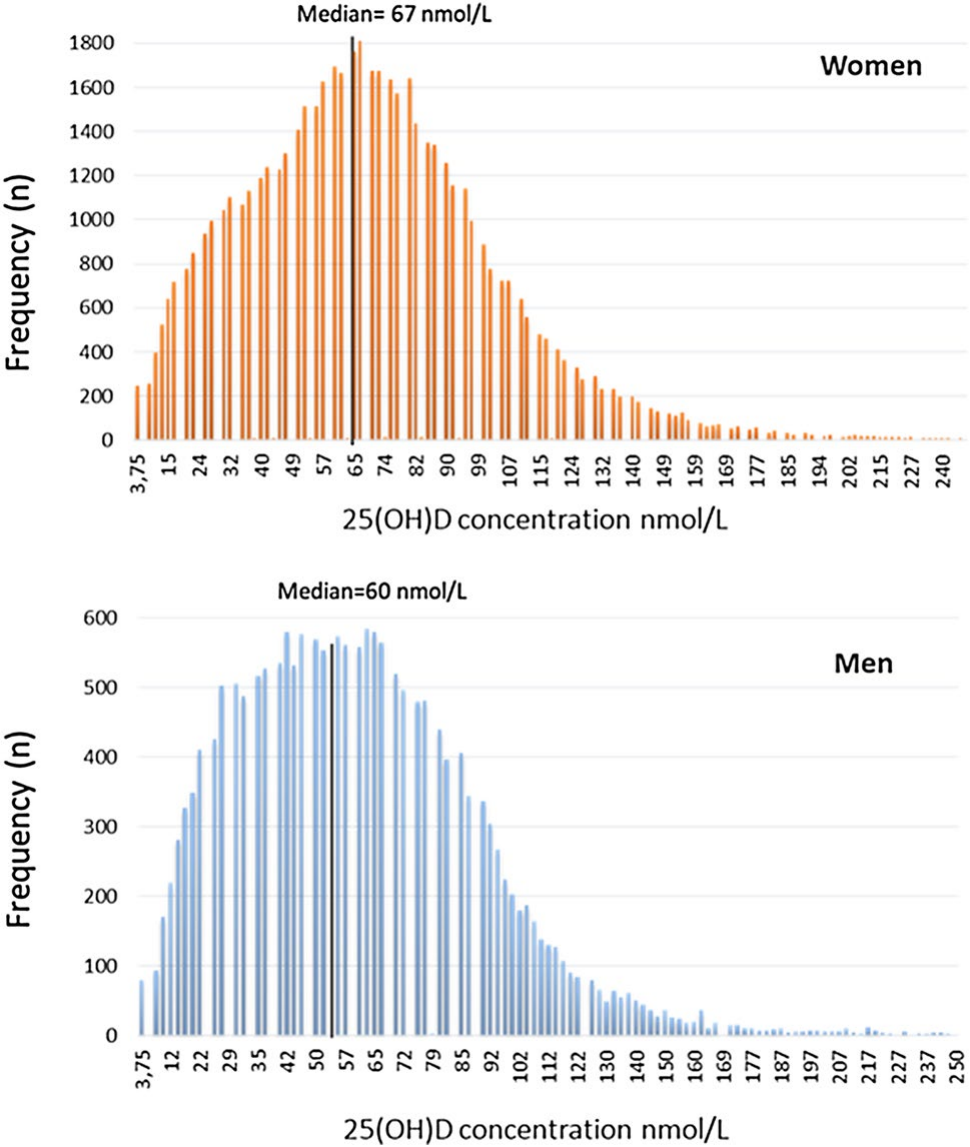
Frequency distribution of serum 25(OH)D concentrations in 18,811 women and 55,424 men from South Tyrol

**Fig. 2:**
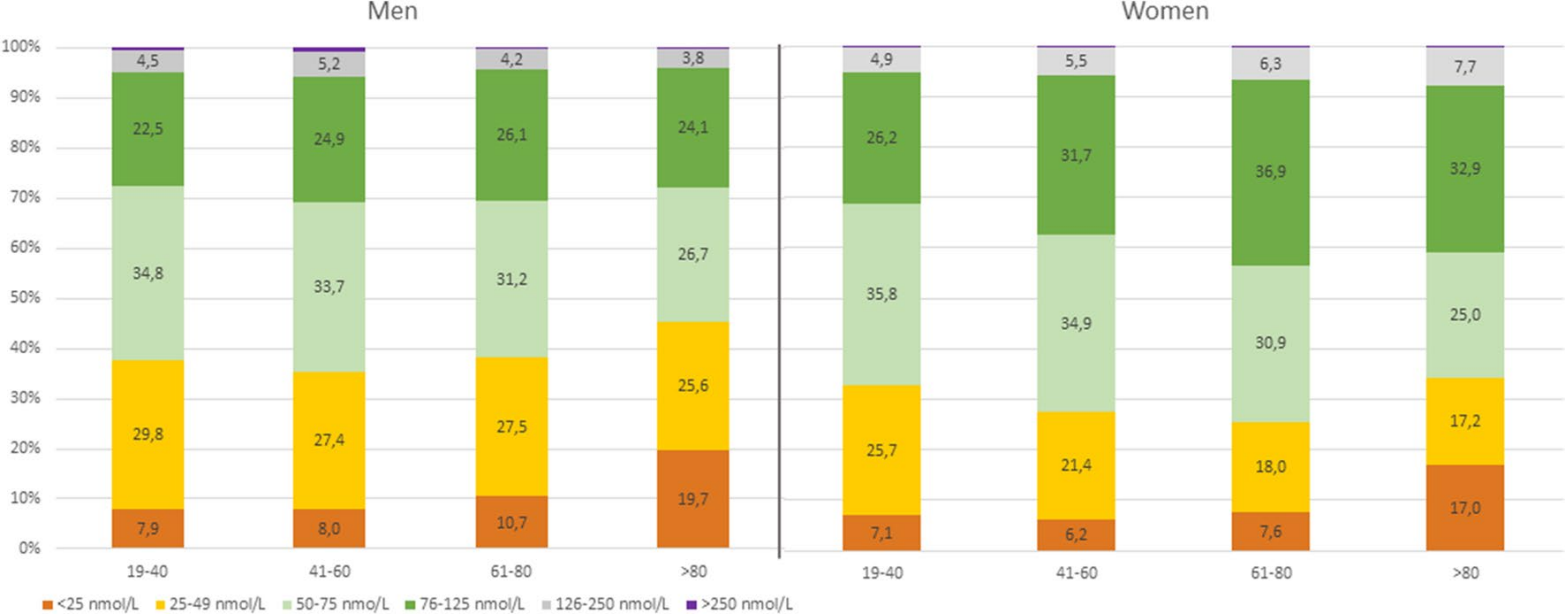
Distribution of serum 25(OH)D concentrations in males and females of different age groups according to the categories recommended by the guidelines of the Endocrine Society Guideline

In males, the 25(OH)D concentration was significantly lower than in females (median 60 vs. 67 nmol/L, *p* < 0.001). Although there was a median difference of 7 nmol/L between males and females, the 2.5th and 97.5th percentiles were rather similar (Table 1). Figure 1 shows the frequency distribution of total 25(OH)D results in males and females. The classification of 25(OH)D results according to the functional categories suggested by the Endocrine Society and the proportion of 25(OH)D categories by age group is shown in Fig. 2.

The mean total 25(OH)D concentration was lowest in males older than 80 years (Table 1). Amongst women, the average 25(OH)D concentration increased with age. Although men also showed significant differences between age groups no clear trend could be observed. An adequate total 25(OH)D concentration (> 76 nmol/L) was most frequently found in individuals aged 61–80 years (43.5% of all women and 30.6% of all men). Severe 25(OH)D deficiency (< 25 nmol/L) was most common in subjects older than 80 years (19.7% of all men and 17% of all women in this age group).

### Total 25(OH)D status in subjects with normal PTH

Out of 74,235 samples, we identified 3801 cases where PTH was requested at the same occasion. The origin of these requests was widespread and not attributable to a specific doctor or discipline. From these cases, 2,313 showed a normal PTH and thus were included in the analysis. In subjects with normal PTH, the mean serum 25(OH)D concentration was 11 nmol/L higher than in the entire cohort (80 vs. 68.6 nmol/L). The difference was more pronounced in women. The 2.5th and 5th percentiles of total 25(OH)D were comparable to the entire cohort, whereas the 95th and 97.5th percentiles differed by as much as 27 nmol/L (Table 1).

### Seasonal variation of total 25(OH)D

**Fig. 3:**
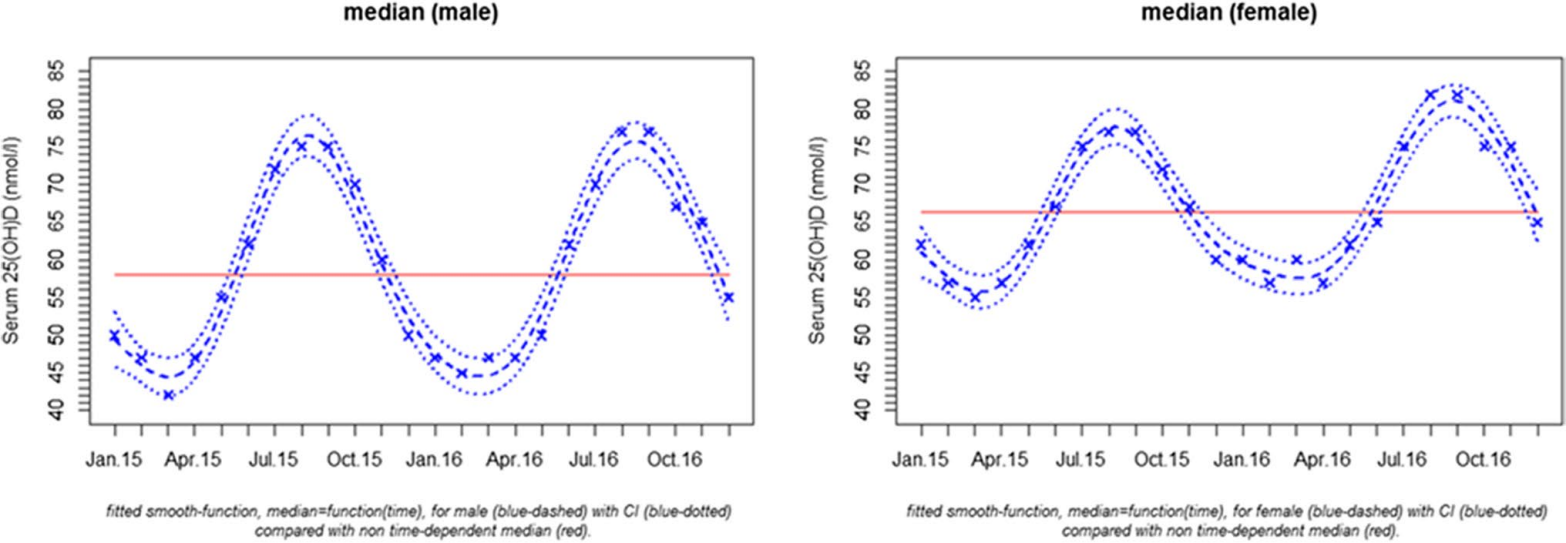
Circannual variation of the median (dashed line) and its 95% confidence interval (dotted line) of serum 25(OH)D in males and females > 18 years of age. A GAM model was used to smoothen the median and to calculate 95% CIs

**Fig. 4:**
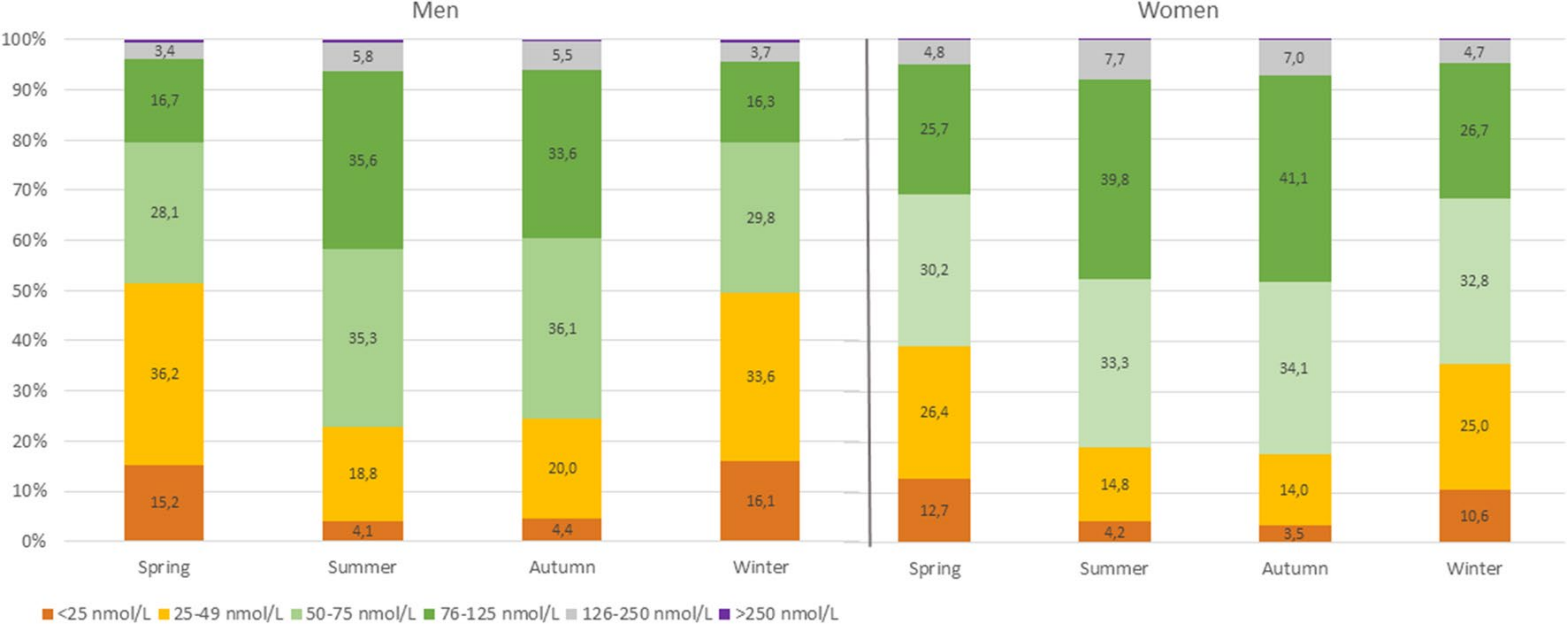
Seasonal distribution of serum 25(OH)D concentrations in males and females according to the categories recommended by the guidelines of the Endocrine Society Guideline

Plotting the median 25(OH)D concentration against the date of blood collection showed a substantial seasonal variation of approximately 20–30% in both sexes with peaks of 25(OH)D in summer and autumn, and nadir between winter and spring (Fig. 3). The season-specific classification of 25(OH)D results according to the functional categories suggested by the Endocrine Society in males and females is presented in Fig. 4 (7). In winter and spring 68.4% of the samples from females and 79.5% of the samples from males showed insufficient 25(OH)D concentrations (< 75 nmol/L). During summer and autumn, the prevalence of insufficient 25(OH)D levels decreased to 52 and 59% for women and men, respectively.

**Fig. 5:**
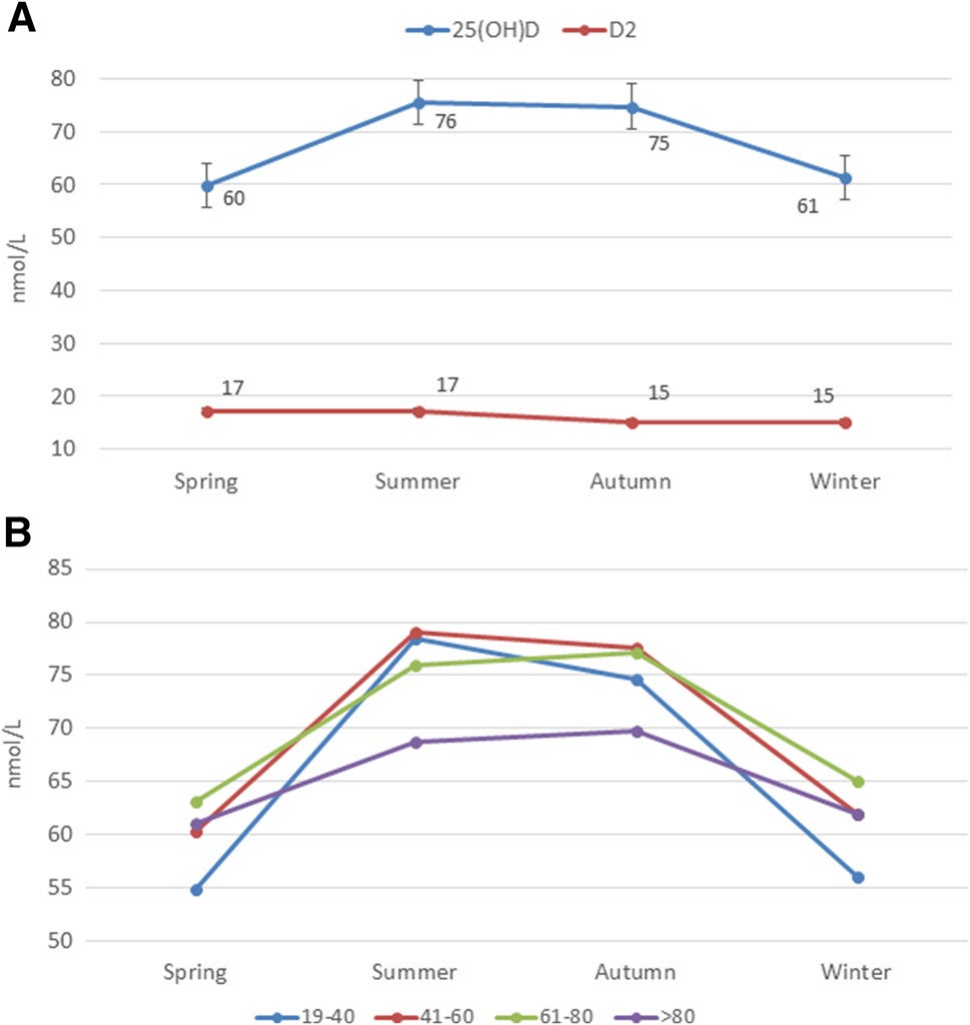
**a** Seasonal variation of mean total serum 25(OH)D and 25(OH)D_2_. **b** Seasonal variation of mean total serum 25(OH)D in different age group

The degree of seasonal variation differed with age. In subjects aged 19–40 years, the maximum seasonal change of serum 25(OH)D was 22.2 nmol/L, whereas in subjects > 80 levels were rather constant (Fig. 5b). As expected, seasonal variation was observed for total 25(OH)D concentration, but not for 25(OH)D_2_ (Fig. 5a).

## Discussion

In South Tyrol, 25(OH)D is measured most frequently in women aged 61–80 years. The average concentration is 68.6 nmol/L with females having higher levels than males. The prevalence of vitamin D deficiency depends on season and gender, but is highest amongst subjects > 80 years of age during winter and spring. 25(OH)D_2_ is measurable in 0.84% of all samples with an average concentration of 17 nmol/L.

The high number of requests in females is supposedly due to an increased awareness of the health issues related to vitamin D deficiency and a more frequent use of supplements. A similar requesting pattern has been reported in the UK [23]. The average 25(OH)D concentration in South Tyrol is close to the sufficiency level and substantially higher than in other European cohorts [24–27]. These differences can be explained by the composition of the study cohorts, the amount of sun exposure, the season of blood collection and the analytical methods used. For example, in 8151 adult Germans, a mean serum 25(OH)D concentration of 45.6 nmol/L was found [28]. This cohort was substantially younger than ours and lived at a latitude between 50° and 51° with approximately 1500 h of sunshine per year. In contrast, South Tyrol is located in the Southern Alps at a latitude of 46° and 47° and has approximately 2000 h of sunshine per year [29]. Furthermore, South Tyrol is a mountainous region with many people living at an altitude between 1000 and 1500 m above sea level where UVB radiation is more intense due to a lower absorption in the atmosphere [30]. In most previous studies, serum 25(OH)D was measured by immunoassays, which may differ systematically in their analytical performance from the NIST 972a aligned LC–MS/MS method used here.

On average South Tyrolean women show a 7 nmol/L higher 25(OH)D concentration than males. Schleicher et al. also observed 4 nmol/L higher 25(OH)D concentrations in white American women than in men [31]. In contrast, other studies showed higher levels in males [9, 21] or no gender difference [28, 32]. These discrepancies might be caused by differences in life style, such as time spent outdoor, clothing and the use of supplements. It can be speculated that differences between males and females are population-specific and there may not be a systemic difference.

25(OH)D_2_ represents a major interference in immunoassays [17, 18]. Our results demonstrate a very low prevalence of quantifiable amounts of 25(OH)D_2_, which is much lower than in France, China and the USA, where vitamin D_2_-containing supplements are frequently used [33]. As in most European countries, vitamin D supplements in Italy contain vitamin D_3_ this difference is not unexpected [33]. Cashman et al. reported age and vitamin D supplementation as positive predictors of 25(OH)D_2_ [20]. Considering that many immunoassays do not detect 25(OH)D_2_ and 25(OH)D_3_ in equimolar fashion laboratories should account for the local prevalence of quantifiable concentrations of 25(OH)D_2_ when choosing their method.

The average PTH concentration decreased continuously across classes of rising 25(OH)D concentrations from 122 pg/mL (25(OH)D: < 25 nmol/L) to 49 pg/mL (25(OH)D: > 250 nmol/L). Interestingly, none of the patients with a serum 25(OH)D concentration > 250 nmol/L showed a suppressed PTH. In subjects with normal PTH, average 25(OH)D was 11 nmol/L higher than in the entire cohort, which alludes to a suboptimal supply with vitamin D in a relevant fraction of our cohort. In 507 subjects, serum calcium measurements were also available. Serum calcium was comparable in subjects with 25(OH)D concentrations < 25 nmol/L (average 2.35 [1.75–2.84] mmol/L) and > 250 nmol/L (2.38 [1.56–2.89] mmol/L). This suggests that the majority of subjects is able to maintain calcium/phosphate homeostasis across a wide range of 25(OH)D concentrations. Furthermore, hypercalcemia does not appear to be a common problem in the presence of excess 25(OH)D concentrations. The lack of clinical information in our cohort precludes an additional evaluation of bone and mineral metabolism. Nevertheless, our results are in agreement with previous studies showing a wide range of 25(OH)D concentrations in different populations with the 5th percentile at approximately ≤ 20 nmol/L. However, in other studies, the 5th percentile was substantially higher [20]. However, all these studies have measured 25(OH)D by immunoassay. At low concentrations, these assays can deviate by more than 10 nmol/L from NIST 972a aligned LC–MS/MS methods [17, 20]. The limited accuracy of immunoassays is mainly due to different efficacies when separating 25(OH)D from VDBP and other carriers. 25(OH)D_2_ and heterophile antibodies are additional interfering factors that can cause inaccuracies [17, 18]. Besides analytical issues, strong regional variations of serum 25(OH)D have also been described [26], which are in large parts driven by variable UV-B exposure, skin color, genetical differences (VDBP) and vitamin D supplementation.

The prevalence of vitamin D deficiency depends on the cut-off used and the season of blood collection. During summer/autumn, 23% of males and 18% of females have deficient 25(OH)D concentrations (< 50 nmol/L). This increases by approximately 40–50% during winter and spring. Previous studies from Italy and other European countries reported a substantially higher prevalence of vitamin D deficiency ranging from 18.6 to 76.1% [20]. In addition, the percentage of results in the adequate range > 75 nmol/L is considerably higher in South Tyrol than in other regions. The high degree of sun exposure is probably a major driver of the rather good 25(OH)D status in South Tyrol. However, analytical issues may also have contributed to these differences.

Variable exposure to UV-B irradiation throughout the year strongly influences circulating 25(OH)D concentrations. In the present study, seasonal variation of serum 25(OH)D was 20 nmol/L in males and 15 nmol/L in females. With increasing age the seasonal variation of 25(OH)D decreases and disappears almost completely in individuals > 80 years. This phenomenon can probably be explained by more sun exposure in younger individuals when compared to older subjects who live predominantly indoor and wear long clothes. In addition, aged skin is less effective in synthesizing vitamin D_3_ [34]. However, seasonal variation of 25(OH)D differs substantially between studies [35–37]. 25(OH)D_2_, representing predominantly oral intake, varies little throughout the year.

Despite a large number of measurements, the present results are limited by an unknown number of repeat analyses in our cohort. In addition, the present cohort includes some vitamin D-supplemented subjects and individuals with renal impairment. However, the very large number of samples most likely compensates these shortcomings. Strict quality control standards and successful participation in an external quality assurance program where target values are aligned to the reference method lend particular strength to our results. Other epidemiologic studies from the USA, Norway and Netherlands that measured 25(OH)D by NIST 972a aligned LC–MS/MS methods found comparable 25(OH)D concentrations [20, 31].

In conclusion, average serum 25(OH)D in South Tyrol is higher than in other European cohorts. The majority of subjects is able to maintain normal calcium and PTH concentrations across a wide range of 25(OH)D concentrations. Seasonal variation is an important aspect in young and middle-aged adults, but becomes less relevant in elderly subjects. 25(OH)D_2_ is of minor practical importance in South Tyrol.

## Electronic supplementary material

Below is the link to the electronic supplementary material.

Supplementary material 1 (DOCX 17 KB)

## References

[1] Giovannucci E, Liu Y, Rimm EB, Hollis BW, Fuchs CS, Stampfer MJ, Willett WC (2006). Prospective study of predictors of vitamin D status and cancer incidence and mortality in men. Natl Cancer Inst.

[2] Mitri J, Muraru MD, Pittas AG (2011). Vitamin D and type 2 diabetes: a systematic review. Eur J Clin Nutr.

[3] Pittas AG, Lau J, Hu FB, Dawson-Hughes B (2007). The role of vitamin D and calcium in type 2 diabetes. A systematic review and meta-analysis. J Clin Endocrinol Metab.

[4] Visser M, Deeg DJ, Lips P, Longitudinal Aging Study Amsterdam (2003). Low vitamin D and high parathyroid hormone levels as determinants of loss of muscle strength and muscle mass (sarcopenia): the Longitudinal Aging Study Amsterdam. J Clin Endocrinol Metab.

[5] Herrmann M, Sullivan DR, Veillard AS, McCorquodale T, Straub IR, Scott R, Laakso M, Topliss D, Jenkins AJ, Blankenberg S, Burton A, Keech AC, FIELD Study Investigators (2015). Serum 25-hydroxyvitamin D: a predictor of macrovascular and microvascular complications in patients with type 2 diabetes. Diabetes Care.

[6] Agmon-Levin N, Theodor E, Segal RM, Shoenfeld Y (2013). Vitamin D in systemic and organ-specific autoimmune diseases. Clin Rev Allergy Immunol.

[7] Holick MF, Binkley NC, Bischoff-Ferrari HA, Gordon CM, Hanley DA, Heaney RP, Murad MH, Weaver CM, Endocrine Society (2011). Evaluation, treatment, and prevention of vitamin D deficiency: an endocrine society clinical practice guideline. J Clin Endocrinol Metab.

[8] Ross AC, Manson JE, Abrams SA, Aloia JF, Brannon PM, Clinton SK, Durazo-Arvizu RA, Gallagher JC (2011). The 2011 report on dietary reference intakes for calcium and vitamin D from the Institute of Medicine: what clinicians need to know. J Clin Endocrinol Metab.

[9] Aloia JF (2011). Report on dietary reference intake for vitamin D: where do we go from here?. J Clin Endocrinol Metab.

[10] Ross AC, Taylor CR, Yaktine AL, Del Valle HB (2011). Committee to review dietary reference intakes for vitamin D and calcium and institute of medicine, dietary reference intakes for calcium and vitamin D.

[11] Soares L, Pedrosa W, Elói-Santos SM, Vasconcellos LS (2017). 25-Hydroxyvitamin D threshold values should be age-specific. Clin Chem Lab Med.

[12] Yetley EA (2008). Assessing the vitamin D status of the US population. Am J Clin Nutr.

[13] Powe CE, Karumanchi SA, Thadhani (2014). Vitamin D-binding protein and vitamin D in blacks and whites. N Engl J Med.

[14] Berg AH, Powe CE, Evans MK, Wenger J, Ortiz G, Zonderman AB, Suntharalingam P, Lucchesi K, Powe NR, Karumanchi SA, Thadhani RI (2015). 24,25-Dihydroxyvitamin d3 and vitamin D status of community-dwelling black and white Americans. Clin Chem.

[15] Valcour A, Blocki F, Hawkins DM, Rao SD (2012). Effects of age and serum 25-OH-vitamin D on serum parathyroid hormone levels. J Clin Endocrinol Metab.

[16] Farrell CJ, Martin S, McWhinney B, Straub I, Williams P, Herrmann M (2012). State-of-the-art vitamin D assays: a comparison of automated immunoassays with liquid chromatography-tandem mass spectrometry methods. Clin Chem.

[17] Farrell C, Soldo J, Williams P, Herrmann M (2012). 25-Hydroxyvitamin D testing: challenging the performance of current automated immunoassays. Clin Chem Lab Med.

[18] Cavalier E, Wallace AM, Carlisi A, Chapelle JP, Delanaye P, Souberbielle JC (2011). Cross-reactivity of 25-hydroxy vitamin D2 from different commercial immunoassays for 25-hydroxy vitamin D: an evaluation without spiked samples. Clin Chem Lab Med.

[19] Woitge HW, Knothe A, Witte K, Schmidt-Gayk H, Ziegler R, Lemmer B, Seibel MJ (2000). Circannual rhythms and interactions of vitamin D metabolites, parathyroid hormone, and biochemical markers of skeletal homeostasis: a prospective study. J Bone Miner Res.

[20] Cashman KD, Dowling KG, Škrabáková Z, Gonzalez-Gross M, Valtueña J, De Henauw S, Moreno L, Damsgaard CT (2016). Vitamin D deficiency in Europe: pandemic?. Am J Clin Nutr.

[21] Tai SS, Bedner M, Phinney KW (2010). Development of a candidate reference measurement procedure for the determination of 25-hydroxyvitamin D3 and 25-hydroxyvitamin D2 in human serum using isotope-dilution liquid chromatography-tandem mass spectrometry. Anal Chem.

[22] Binkley N, Sempos CT (2014). Vitamin D standardization program (VDSP). standardizing vitamin D assays: the way forward. J Bone Miner Res.

[23] Zhao S, Gardner K, Taylor W, Marks E, Goodson N (2015). Vitamin D assessment in primary care: changing patterns of testing. Lond J Prim Care (Abingdon).

[24] Carnevale V, Modoni S, Pileri M, Di Giorgio A, Chiodini I, Minisola S, Vieth R, Scillitani A (2001). Longitudinal evaluation of vitamin D status in healthy subjects from southern Italy: seasonal and gender differences. Osteoporos Int.

[25] Adami S, Romagnoli E, Carnevale V, Scillitani A, Giusti A, Rossini M, Gatti D, Nuti R, Minisola S, Italian Society for Osteoporosis, Mineral Metabolism and Bone Diseases (SIOMMMS) (2011). Guidelines on prevention and treatment of vitamin D deficiency. Italian Society for Osteoporosis, Mineral Metabolism and Bone Diseases (SIOMMMS). Reumatismo.

[26] Hilger J, Friedel A, Herr R, Rausch T, Roos F, Wahl DA, Pierroz DD, Weber P, Hoffmann K (2014). A systematic review of vitamin D status in populations worldwide. Br J Nutr.

[27] Spiro A, Buttriss JL (2014). Vitamin D: an overview of vitamin D status and intake in Europe. Nutr Bull.

[28] Rabenberg M, Scheidt-Nave C, Busch MA, Rieckmann N, Hintzpeter B, Mensink GB (2015). Vitamin D status among adults in Germany–results from the German Health Interview and Examination Survey for Adults (DEGS1). BMC Public Health.

[29] http://dati.retecivica.bz.it/it/dataset/irraggiamento-solare-annuale-medio-sulle-principali-aree-urbanedellalto-adige-in-wh-m-progetto

[30] Holick MF, Chen TC, Lu Z, Sauter E (2007). Vitamin D and skin physiology: a D-lightful story. J Bone Miner Res.

[31] Schleicher RL, Sternberg MR, Looker AC, Yetley EA, Lacher DA, Sempos CT, Taylor CL, Durazo-Arvizu RA, Maw KL, Chaudhary-Webb M, Johnson CL, Pfeiffer CM (2016). National estimates of serum total 25-hydroxyvitamin D and metabolite concentrations measured by liquid chromatography-tandem mass spectrometry in the US population during 2007–2010. J Nutr.

[32] Hintzpeter B, Mensink GB, Thierfelder W, Müller MJ, Scheidt-Nave C (2008). Vitamin D status and health correlates among German adults. Eur J Clin Nutr.

[33] Armas LA, Hollis BW, Heaney RP (2004). Vitamin D2 is much less effective than vitamin D3 in humans. J Clin Endocrinol Metab.

[34] Gennari C (2001). Calcium and vitamin D nutrition and bone disease of the elderly. Public Health Nutrb.

[35] Jablonski NG, Chaplin G (2018). The roles of vitamin D and cutaneous production in human evolution and health. Int J Paleopathol.

[36] Khoo AL, Koenen HJ, Chai LY, Sweep FC, Netea MG, van der Ven AJ, Joosten I (2012). Seasonal variation in vitamin D3 levels is paralleled by changes in the peripheral blood human T Cell compartment. PLoS One.

[37] Bose S, Breysse PN, McCormack MC, Hansel NN, Rusher RR, Matsui E, Peng R, Curtin-Brosnan R, Diette GB, Center for Childhood Asthma in the Urban (2013). Outdoor exposure and vitamin D levels in urban children with asthma. Nutr J.

