# Supplementary material for: LC–MS/MS based 25(OH)D status in a large Southern European outpatient cohort: gender- and age-specific differences
Source: Eur J Nutr. 2018 Aug 7;58(6):2511–20. doi: 10.1007/s00394-018-1803-1 (PMC6689275; doi:10.1007/s00394-018-1803-1)
Supplement: Supplementary file 1 — Supplementary material 1 (DOCX 17 KB) [file 394_2018_1803_MOESM1_ESM.docx]

**Supplementary Table 1:** Prevalence of serum 25(OH)D_2_ in 74,235 serum samples from South Tyrol – descriptive statistics.

|  | All | | | 19-40 YEARS | | | 41-60 YEARS | | 61-80 YEARS | | | >80 YEARS | |
| --- | --- | --- | --- | --- | --- | --- | --- | --- | --- | --- | --- | --- | --- |
| 25(OH)D2, nmol/L | | | | | | | | | | | | | |
| Sex | M  (n=66) | F  (n=554) | M  (n=4) | | | F  (n=21) | M  (n=16) | F  (n=97) | M  (n=25) | F  (n=281) | | M  (n=21) | F  (n=155) |
| Median | 13.5 | 17 | 12.5 | | 12 | | 12 | 10 | 12 | | 17 | 15 | 20 |
| SD | 7.9 | 25.5 | 4.8 | | 10.6 | | 3.6 | 9.8 | 8.9 | | 26.54 | 9.1 | 29.1 |
